# Terpinen-4-ol and nystatin co-loaded precursor of liquid crystalline system for topical treatment of oral candidiasis

**DOI:** 10.1038/s41598-020-70085-z

**Published:** 2020-07-31

**Authors:** Renata Serignoli Francisconi, Patricia Milagros Maquera-Huacho, Caroline Coradi Tonon, Giovana Maria Fioramonti Calixto, Janaína de Cássia Orlandi Sardi, Marlus Chorilli, Denise Madalena Palomari Spolidorio

**Affiliations:** 1 Department of Physiology and Pathology, School of Dentistry of Araraquara, Universidade Estadual Paulista, UNESP, Araraquara, SP Brazil; 2 Department of Drugs and Medicines, School of Pharmaceutical of Araraquara, UNESP, Araraquara, SP Brazil; 3 Department of Physiological Sciences, School of Dentistry of Piracicaba, UNICAMP, Piracicaba, SP Brazil

**Keywords:** Biofilms, Fungi

## Abstract

This study was performed to develop a liquid crystalline system (LCS) incorporated with terpinen-4-ol and nystatin to evaluate its antifungal, antibiofilm, and synergistic/modulatory activity against *Candida albicans*. The LCS was composed of a dispersion containing 40% propoxylated and ethoxylated cetyl alcohol, 40% oleic acid, and 0.5% chitosan dispersion. According to analysis by polarized light microscopy, rheology, and mucoadhesion studies, the incorporation of 100% artificial saliva increased the pseudoplasticity, consistency index, viscosity, and mucoadhesion of the formulation. The minimum inhibitory concentration, minimum fungicidal concentration, and rate of biofilm development were used to evaluate antifungal activity; the LCS containing terpinen-4-ol and nystatin effectively inhibited *C. albicans* growth at a lower concentration, displaying a synergistic action. Therefore, LCS incorporated with terpinen-4-ol and nystatin is a promising alternative for preventing and treating infections and shows potential for the development of therapeutic strategies against candidiasis.

## Introduction

*Candida* species are pathogenic yeasts and the leading causes of several major fungal infections in humans. These infections can be caused by increased use of immunosuppressive therapies, acquired immunodeficiency syndrome, and the emergence of drug resistance^1,2^. *Candida albicans* is the most commonly isolated yeast species in the oral microbiota and may cause infections in the presence of other associated factors such as vertical dimension reduction, unstable occlusion, old prostheses, and roughness causing local or systemic infection^3^. Among the different virulence factors, the initial adhesion resulting in biofilm formation on abiotic and biotic surfaces plays a critical role in the pathogenesis of *C. albicans*.

Several antifungal agents are used to prevent and treat oral candidiasis, such as polyenes, ketoconazole, and miconazole^4^. However, the clinical presentation of less susceptible strains leads to treatment failure, increasing disease recurrence. Several mechanisms of azole-resistant species have been reported, including those with changes in the cell wall or plasma membrane to impair azole uptake; overexpression or mutations in the target enzyme of azoles; and efflux of drugs mediated by membrane transport proteins^5^. Resistance appears to increase proportionally with the extent of previous exposure to antifungal drugs. In addition, studies have shown some medications may cause injuries in the kidney and liver^6^, highlighting the need to develop new therapeutic strategies and search for agents with novel mechanisms of action that may be used independently or in combination with conventional medicines.

Drug resistance is a matter of concern. The search for new agents may lead to the development of new antifungal agents that are effective against biofilms. Although the polyene nystatin is widely used to control and reduce oral fungal infections, polyenes are highly toxic to the host and their prolonged use can lead to drug resistance^7,8^. Traditional medicinal plants and alternative therapies are attractive sources of new antimicrobial agents^9^. Natural products are considered as an important source for the development of new antifungal therapies. *Melaleuca alternifolia* oil, also known as tea tree oil (TTO), is composed mainly of monoterpenes and alcohols. This oil has broad-spectrum activity against Gram-positive and Gram-negative bacteria as well as antimicrobial-resistant and multidrug resistant microorganisms^10^.

Recent evidence has shown that TTO facilitates the maintenance of oral hygiene and prevention of oral diseases^2^. Terpinen-4-ol is the main active compound in TTO and has gained attention for its antimicrobial, antifungal, and anti-inflammatory properties^11^. Terpinen-4-ol induces membrane loss to disrupt the integrity and physiology of microbial cells^12^. Several reports have demonstrated oral care products containing terpinen-4-ol have demonstrable antiseptic effects and inhibit bacterial growth and adhesion to the dental biofilm^12,13^. Low concentrations of terpinen-4-ol do not display toxicity towards fibroblasts and epithelial cells, allowing for topical use with reduced adverse effects^14–17^.

The incorporation of natural products, such as terpinen-4-ol, into a reliable delivery system in a clinical setting is a promising option for treating oral fungal infections. In an adhesive mucosystem, also known as a liquid crystal system (LCS), the drug is incorporated into the system to improve its antimicrobial action and extend the contact time of the preparation at the site of action or absorption, improving the retention time of the drug within the mucosa. Because saliva secretion (0.5–2.0 L/day) can dilute the medication, leading to an inevitable dose reduction^18^, an LCS in the oral cavity can promote the sustained release of active substances and improve the stability of formulations^19,20^.

Based on the emergence of resistance to antifungal agents and reduced effectiveness of current drugs, new therapies incorporating delivery systems have become an important treatment strategy for fungal infections^20^. The additive and synergistic actions of these drugs may lead to increased efficacy and/or reduced resistance, as well as a lower risk of adverse effects for patients.

The objective of this study was to develop an LCS associated with terpinen-4-ol and nystatin to evaluate the antifungal, antibiofilm, and synergistic/modulatory activity of the system.

## Results

### Development of the liquid crystal system (LCS)

**Figure 1:**
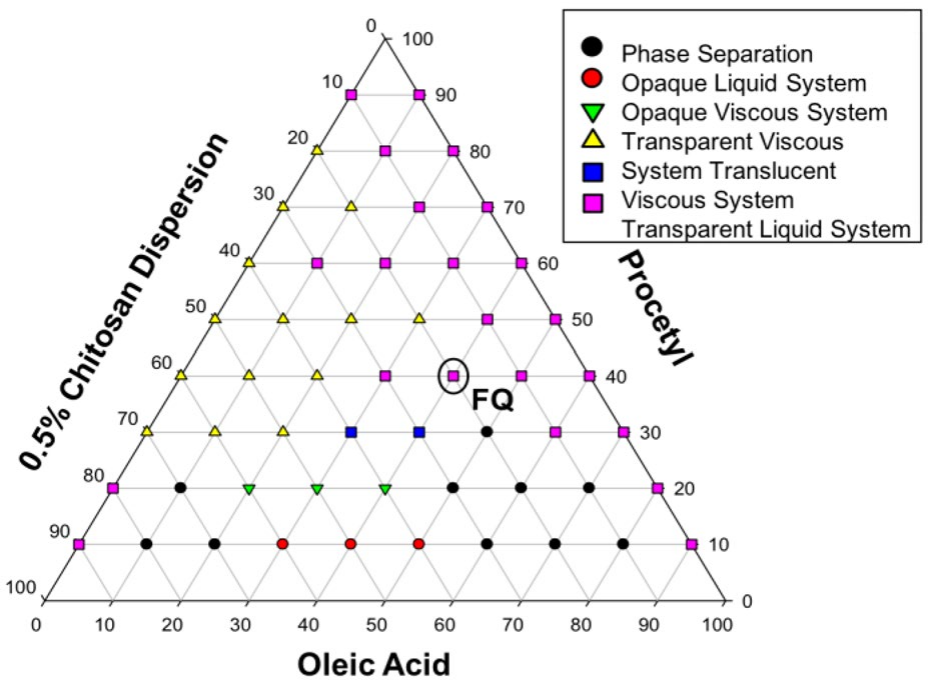
Diagrams composed of Procetyl (PRO), oleic acid (OA) and 0.5% chitosan dispersion. FQ is the selected liquid crystal precursor system (LCS) consisting of 40% surfactant, 40% oil phase and 20% aqueous phase.

A phase diagram, composed of PPG-5-CETETH-20, OA, and 0.5% QS, is shown in Fig. 1. The diagram shows that the formulations were established using 40% surfactant, and that the LCS precursor formulation (FC) shown in the diagram, consisting of 40% surfactant, 40% oil phase, and 20% aqueous phase, showed the greatest potential for use as the LCS for oral applications.

### Physicochemical characterization

**Figure 2:**
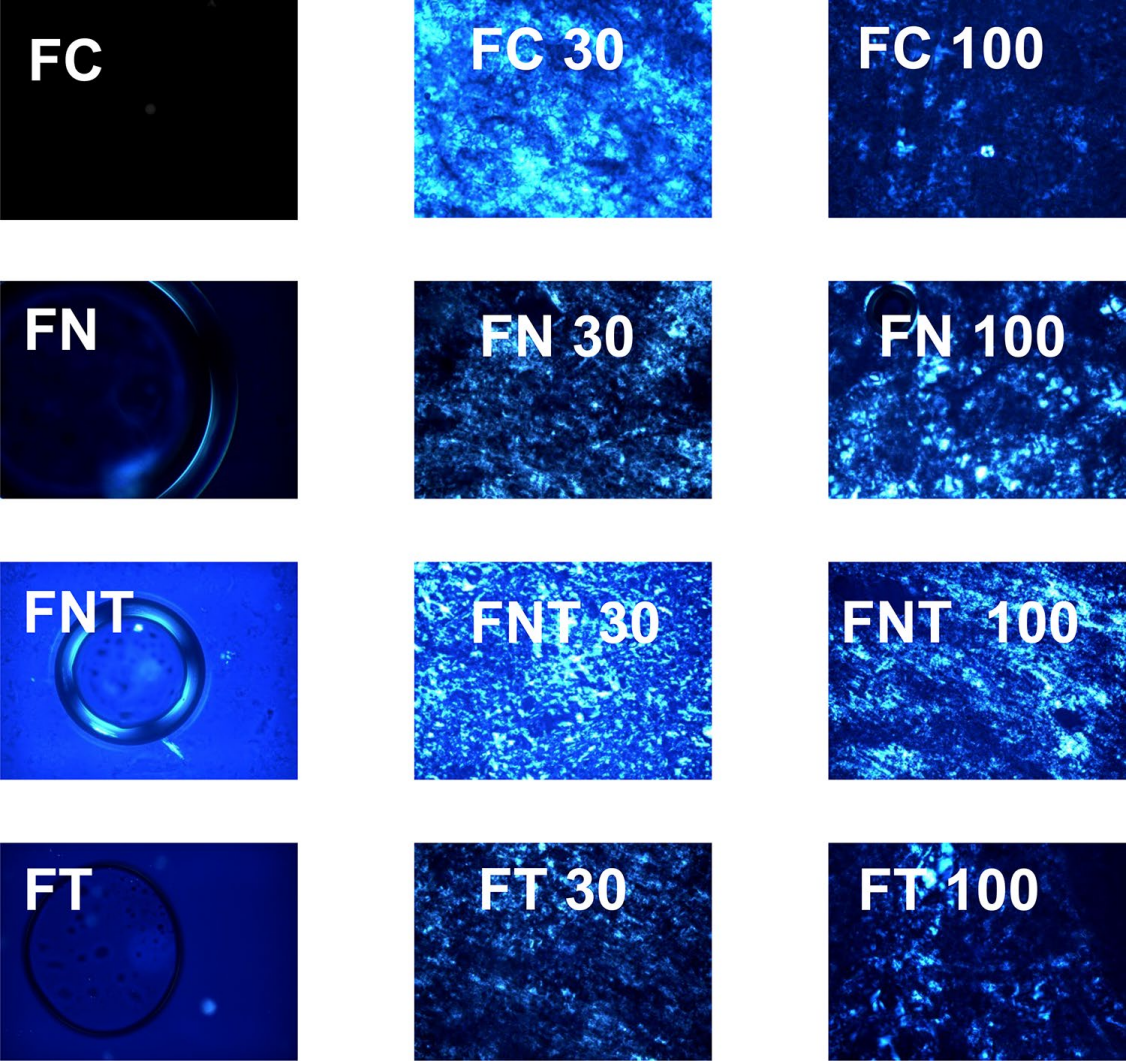
Photomicrographs representing the effect of artificial saliva on the LCS structure. Increase ×20. FC: LCS formulation, FN: LCS with nystatin; TNF: LCS with nystatin and terpinen-4-ol; FT: LCS with terpinen-4-ol, 30 and 100: dilution of F containing respectively 30 and 100% artificial saliva.

Figure 2 shows the photomicrograph results of the formulations after adding 30% and 100% artificial saliva. The addition of artificial saliva resulted in formation of a lamellar liquid crystalline mesophase and hexagonal liquid crystalline mesophase, as identified by Malta crosses and streaking, respectively. LCS precursor formulation (FC), LCS precursor with terpinen-4-ol (FT), LCS precursor with nystatin (FN), and LCS precursor with nystatin and terpinen-4-ol (FNT) groups present dark field with low viscosity, showing microemulsified system-like behaviors. Addition of 30% and 100% artificial saliva to the formulations altered their liquid crystalline structures. The FC, FN, FT, and FNT groups behaved as LCS precursors. Photomicrographs showed that formulations containing terpinen-4-ol and nystatin (FT, TNF, FN, FN30, FT30, FNT30, FN100, FT100, and FNT100) did not cause structural modifications.

**Figure 3:**
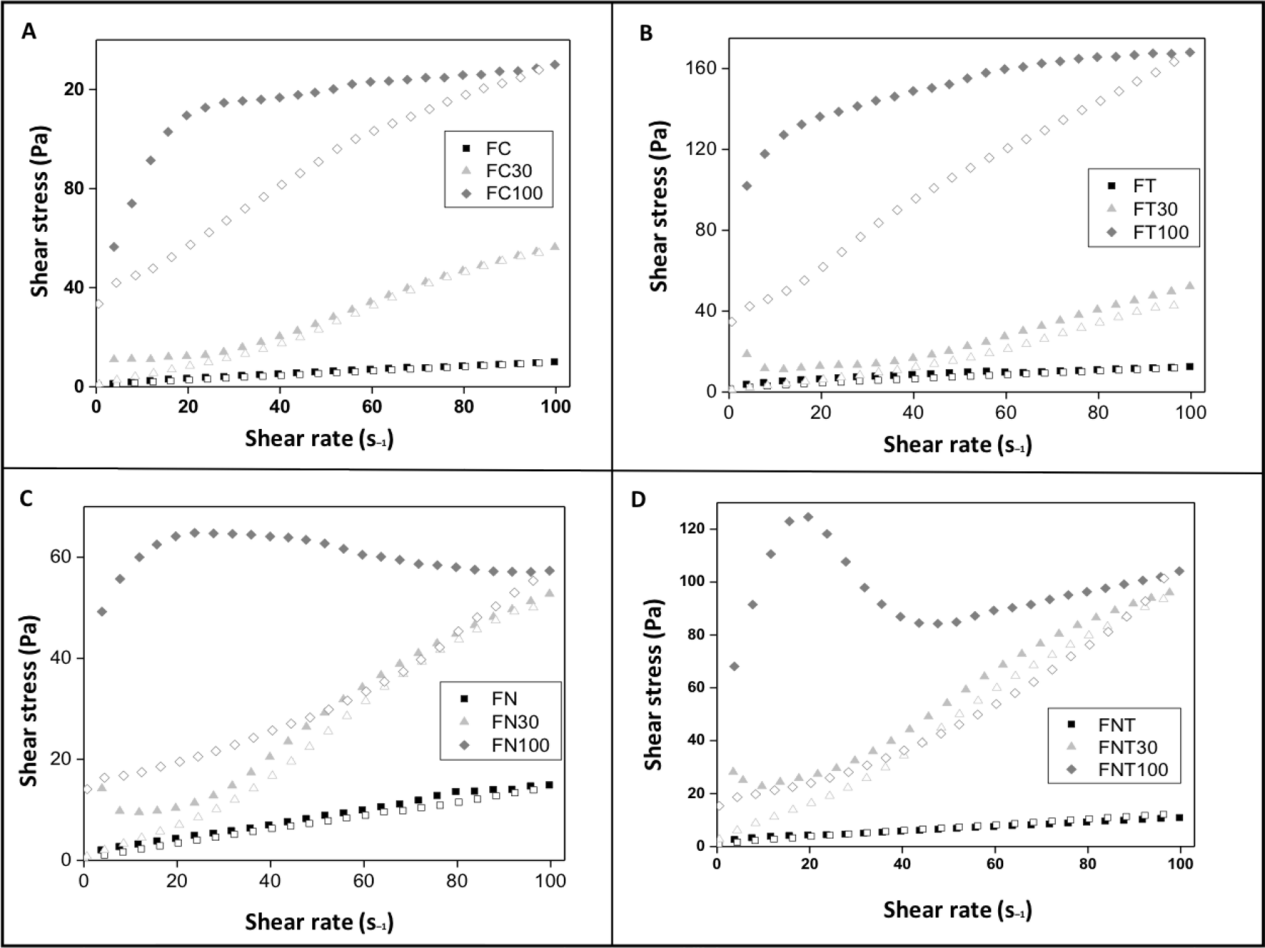
Flow rheogram of the formulations. Closed symbols represent the upward curve and open symbols represent the downward curve. (**A**) Represents the different groups of the LCS formulation; (**B**) LCS with terpinen-4-ol; (**C**) LCS and nystatin; (**D**) LCS with terpinen-4-ol and nystatin.

The relationships between the shear stress and shear rate for all formulations are shown in Fig. 3. The obtained formulations behaved as pseudoplastic fluids (n < 1); the viscosity of the fluids decreased when the shear rate increased because the orientation of the rigid particles within the formulation occurred in the direction of flow, generating shear thinning. However, when this shearing stopped, the formulation regained its initial viscosity. Thus, the FC, FT, FN, and FNT groups showed low viscosity; however, upon adding 30% and 100% artificial saliva, the viscosity increased. The values for the consistency index (K) and flow rate (n) obtained using Eq. (1) are shown in Table 1. Addition of 100% artificial saliva decreased the flow rate (n) in all formulations, increased their pseudoplasticity and consistency index, and increased their viscosity. In addition, all formulations with 100% artificial saliva were found to display thixotropy, revealing that formulations forming rigid structures took a longer time to regain their original conformations when shearing forces were removed.

**Table 1:** FC: LCS formulation; FN: LCS with nystatin; TNF: LCS with nystatin and terpinen-4-ol; FT: LCS with terpinen-4-ol; 30 and 100: percentages of artificial saliva in the crystalline liquid system.

Formulations	Flow rate (n)	Consistency index (k)	
FC	0.66 ± 0.01		0.48 ± 0.02
FC30	0.99 ± 0.05		0.58 ± 0.13
FC100	0.18 ± 0.02		59.0 ± 3.69
FT	0.37 ± 0.01		2.17 ± 0.09
FT30	1.02 ± 0.09		0.46 ± 0.18
FT100	0.14 ± 0.00		87.4 ± 0.98
FN	0.78 ± 0.02		0.41 ± 0.04
FN30	0.96 ± 0.05		0.66 ± 0.16
FN100	0.01 ± 0.01		58.5 ± 3.45
FNT	0.56 ± 0.03		0.78 ± 0.08
FNT30	0.78 ± 0.05		2.68 ± 0.59
FNT100	0.01 ± 0.03		97.6 ± 12.0

**Figure 4:**
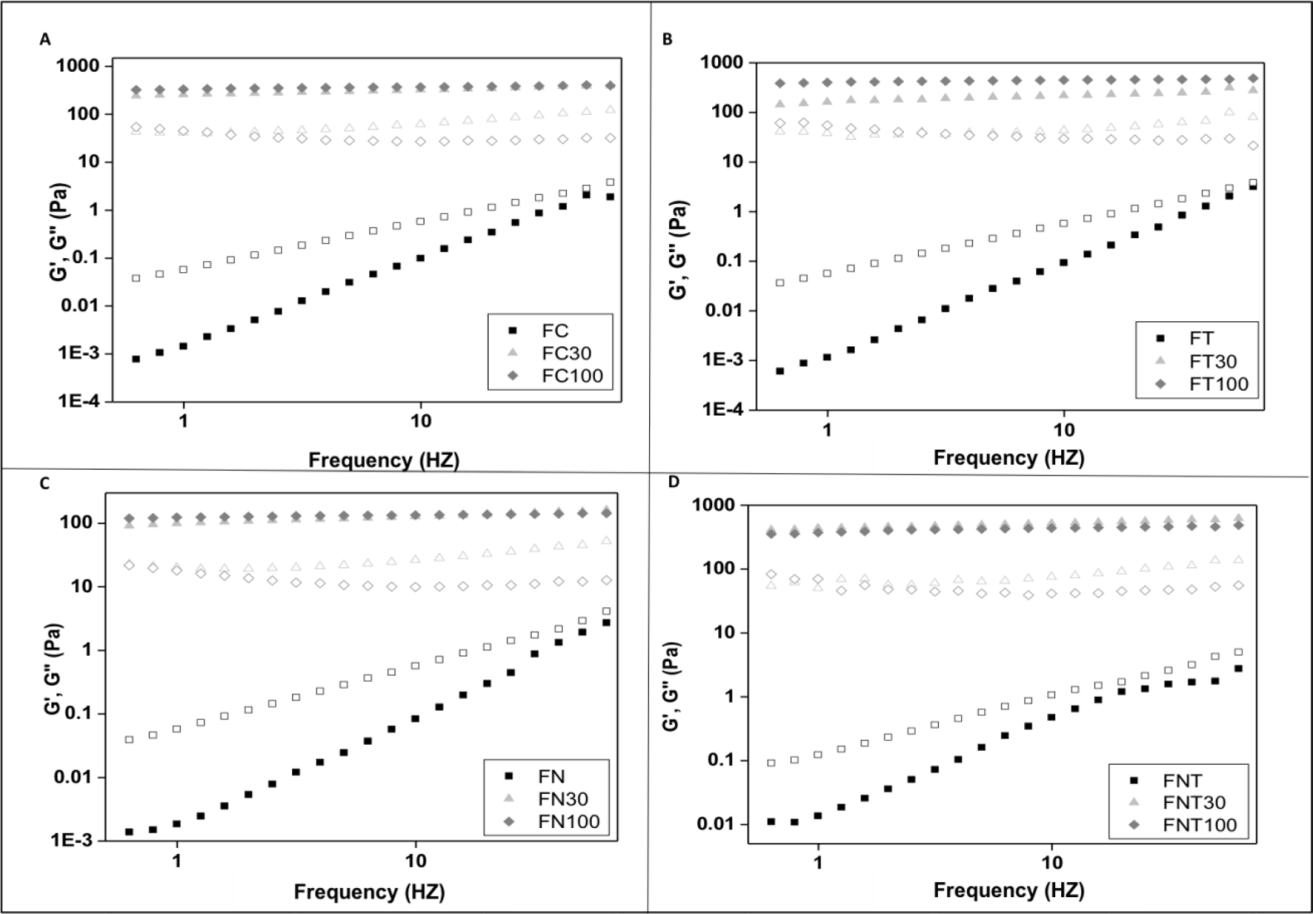
Variation of the storage modulus G′ (full symbols) and loss G″ (empty symbols) as a function of the frequency of the formulations. (**A**) Represents the different groups of the LCS; (**B**) LCS with terpinen-4-ol; (**C**) LCS and nystatin; (**D**) LCS with terpinen-4-ol and nystatin.

Figure 4 shows the temporal evolution of the elastic modulus (G′) and viscous modulus (G″) as a function of the applied frequency. The magnitude of these moduli is a qualitative indication of the structure of the system; for example, if G′ >  > G″, the system is chemically interconnected, while if G′ > G″, the system is structured by connections; furthermore, if G′ ≤ G″, the molecules of the system are considered as bound only by physical interactions.

The results showed that all formulations without artificial saliva and all formulations with 30% artificial saliva were more viscous than elastic (G″ > G′). However, addition of 100% artificial saliva caused all formulations to be more elastic than viscous (G′ > G″), with highly organized gel structures.

**Figure 5:**
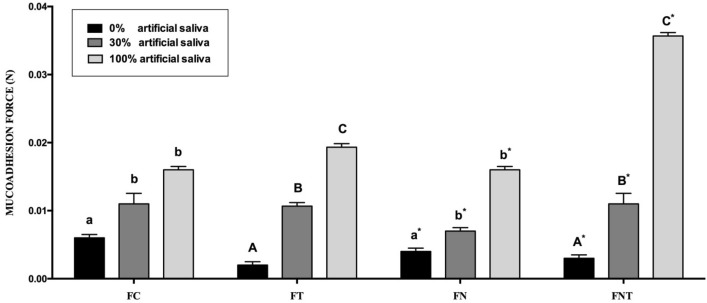
Mucoadhesion (N) of the formulations. FC: LCS formulation; FT: LCS with terpinen-4-ol; FN: LCS with nystatin; FNT: LCS with nystatin and terpinen-4-ol; 30 and 100: dilution of F containing respectively 30 and 100% artificial saliva. *p < 0.05.

The forces necessary to remove the analyzed formulations from the surface of the mucosa are shown in Fig. 5. The addition of artificial saliva to the system significantly increased the mucoadhesion of the formulations because of the formation of the LCS.

### Minimum inhibitory concentration (MIC) and minimum fungicidal concentration (MFC)

The MIC and MFC values of terpinen-4-ol, nystatin, and LCS associated with the test solutions on planktonic *C. albicans* cultures are shown in Table 2. Concentrations of 4.53 mg/mL terpinen-4-ol and 0.004–0.16 mg/mL nystatin inhibited the growth of planktonic *C. albicans* cultures. The association of LCS with the test solutions was highly effective at low concentrations on planktonic cultures (1.06 mg/mL terpinen-4-ol and 0.0001–0.00025 mg/mL nystatin).

**Table 2:** Antifungal activity of terpinen-4-ol, nystatin and LCS associated on planktonic *Candida albicans* cultures (mg/mL).

*Candida albicans*						
	Genotype A		Genotype B		SC 5314	
	CIM	CFM	CIM	CFM	CIM	CFM
T-4-ol	4.53	4.53	4.53	4.53	4.53	4.53
T-4-ol + LCS	1.06	4.53	1.06	2.31	1.06	4.53
Nystatin	0.008	0.016	0.008	0.016	0.004	0.004
Nystatin + LCS	0.00025	0.0005	0.00025	0.0001	0.0005	0.0005

### Biofilm formation

The effects of terpinen-4-ol and nystatin on *C. albicans* biofilms were evaluated (Table 3). Terpinen-4-ol (4.53 mg/mL) inhibited all *C. albicans* biofilms. However, nystatin was effective at 0.004–0.128 mg/mL. The LCS was effective at lower concentrations (1.06–2.31 mg/mL terpinen-4-ol and 0.00025–0.032 mg/mL nystatin) and successfully inhibited the growth of *C. albicans*.

**Table 3:** Antifungal activity of terpinen-4-ol, nystatin and LCS associated on *Candida albicans* biofilms (mg/mL).

*Candida albicans*						
	Genotype A		Genotype B		SC 5,314	
	CIM	CFM	CIM	CFM	CIM	CFM
T-4-ol	4.53	4.53	4.53	4.53	4.53	4.53
T-4-ol + LCS	2.31	4.53	2.31	4.53	1.06	4.53
Nystatin	0.128	0.064	0.128	0.064	0.004	0.008
Nystatin + LCS	0.032	0.128	0.032	0.128	0.0005	0.004

In general, the association of LCS with terpinen-4-ol or nystatin showed the strongest effect on planktonic cultures and *C. albicans* biofilms for both test solutions.

Planktonic cultures and biofilms were evaluated at the lowest concentrations of each test solutions able to completely inhibit growth. Next, the terpinen-4-ol and nystatin MIC values were converted to fractional inhibitory concentration (FICs) and evaluated by the checkerboard method*.* A synergistic effect of the test solutions was observed in planktonic and biofilm cultures. Synergistic effects were observed at 1.06 and 0.57 mg/mL of terpinen-4-ol and 0.002 and 0.001 mg/mL of nystatin in planktonic cultures (Table 4). Additionally, additive effects were observed at 0.57 mg/mL of terpinen-4-ol/LCS and 0.00012–0.0003 mg/mL of nystatin/LCS (Table 5).

**Table 4:** Synergistic/modulating activity of terpinen-4-ol and nystatin on planktonic culture of *Candida albicans* (mg/mL).

Planktonic culture	Terpinen-4-ol + Nystatin			Effect
	Terpinen-4-ol	Nystatin	FICI	
	2.31	0.002	0.76	Additive
Genotype A	2.31	0.001	0.63	Additive
	2.31	0.0005	0.57	Additive
	2.31	0.00025	0.54	Additive
	1.06	0.004	0.73	Additive
	1.06	0.002	0.48	Synergistic
	0.57	0.004	0.63	Additive
	0.57	0.002	0.38	Synergistic
	2.31	0.002	0.76	Additive
Genotype B	1.06	0.004	0.73	Additive
	1.06	0.002	0.48	Synergistic
	0.57	0.004	0.63	Additive
	2.31	0.001	0.76	Additive
SC 5314	1.11	0.002	0.75	Additive
	1.11	0.001	0.50	Synergistic
	0.57	0.002	0.63	Additive

**Table 5:** Synergistic/modulating activity of terpinen-4-ol and nystatin associated with LCS on planktonic culture of *Candida albicans* (mg/mL).

Planktonic culture	Terpinen-4-ol + Nystatin			Effect
	T-4-ol + LCS	Nyst + LCS	FICI	
	0.57	0.00012	0.99	Additive
Genotype A	0.57	0.00006	0.75	Additive
	0.57	0.00003	0.63	Additive
	0.57	0.00012	0.99	Additive
Genotype B	0.57	0.00006	0.75	Additive
	0.57	0.00006	1.00	Additive
SC 5314	0.57	0.00003	0.76	Additive

The synergism/additive effect of terpinen-4-ol and nystatin associated with LCS were evaluated. Terpinen-4-ol/LCS showed synergistic effects at 1.06 and 0.57 mg/mL and nystatin/LCS at 0.008–0.064 mg/mL on *C. albicans* biofilms (Table 6).

**Table 6:** Synergistic/modulating activity of terpinen-4-ol and nystatin associated with LCS on biofilm of *C. albicans*.

Biofilm	Terpinen-4-ol + Nystatin			Effect
	T-4-ol + LCS (CIM)	Nyst + LCS (CIM)	FICI	
	2.31	0.064	0.76	Additive
Genotype A	2.31	0.032	0.63	Additive
	2.31	0.016	0.57	Additive
	2.31	0.008	0.51	Additive
	2.31	0.004	0.53	Additive
	2.31	0.002	0.52	Additive
	1.06	0.128	0.73	Additive
	1.06	0.064	0.48	Synergistic
	1.06	0.032	0.36	Synergistic
	1.06	0.016	0.30	Synergistic
	1.06	0.008	0.27	Synergistic
	0.57	0.128	0.63	Additive
	2.31	0.064	0.76	Additive
Genotype B	1.06	0.128	0.73	Additive
	0.57	0.128	0.63	Additive
	2.31	0.064	0.76	Additive
SC 5314	2.31	0.032	0.63	Additive
	2.31	0.016	0.57	Additive
	1.06	0.128	0.73	Additive
	1.06	0.064	0.48	Synergistic
	1.06	0.032	0.36	Synergistic
	0.57	0.128	0.63	Additive
	0.57	0.064	0.38	Synergistic

## Discussion

Current efforts to control the increase microbial resistance have opened new perspectives regarding the use of alternative therapies for fungal control; drug delivery systems have shown promise as an alternative treatment method because they increase drug solubility. In the present study, terpinen-4-ol and nystatin-loaded LCS was developed to investigate its antifungal and antibiofilm activity in resistant *C. albicans* strains, as resistance to antimicrobial drugs is the major obstacle to the treatment of candidiasis. Incorrect medical prescriptions and long hospital stays are the leading causes of azole-resistant isolates in invasive infections^21,22^. The use of combined antifungal therapies has enabled the development of drugs with different mechanisms of action to increase antifungal potency. This may be related to the acquisition of mechanisms of resistance, as the isolates were obtained from patients with diabetes^8^. Similarly, Zomorodian et al.^23^ suggested that patients with poor glycemic control were at a higher risk of developing *Candida* infections.

A phase diagram was constructed to determine the percentages of surfactant, oil phase, and aqueous phase in the LCS. Based on the phase diagram, the FC formulation was found to be the most efficient LCS. This is because the liquid phase can be administered by syringe, as it is present in a transition state for viscous systems, characteristic of LCS as the aqueous phase increases. The diagram showed that the formulations were stabilized from 40% surfactant; therefore, the FC formulation consisted of 40% surfactant, 40% oil phase, and 20% aqueous phase was chosen because it formed the LCS with the lowest concentration of surfactant in order to decrease the toxic potential of the formulations^20^. These results are consistent with those of Calixto et al.^20^, who also obtained the transition phase after addition of artificial saliva that formed a formulation that increases bioavailability, improves drug release, and maintains local concentrations in the desired area^24^. After adding artificial saliva, a mucoadhesion increase was observed, which allowed the drug to remain in contact with the target area for a longer time and improve its effectiveness. Because of the volatility of terpinen-4-ol, its application in LCS is an effective and safe alternative to take advantage of its stability and antibiofilm activity against *C. albicans*. In addition, the LCS improved the biodegradability and tolerability of the treatment system, allowing for gradual release and, in many cases, increasing the effectiveness of the treatment^25^.

The obtained formulations were analyzed by polarized light microscopy (PLM), rheology, and mucoadhesion analyses. PLM showed that all formulations had a low viscosity and behaved as micro emulsion systems; when artificial saliva was added, the formulations behaved as LCS precursors. In the development of LCS drug delivery systems, the crystalline liquid phase of the system may influence drug release. Thus, when artificial saliva was added, the proportion of the aqueous phase was increased and the system became a LCS^26^.

Rheological analysis evaluates the deformational behavior of a flowing fluid and characterizes its distribution systems. In this study, the obtained formulations behaved as pseudoplastic fluids. According to Aida et al.^27^, pseudoplastic flow occurs in materials that undergo a decrease in viscosity when the shear rate is increased. The formulations did not present any structural organization that could be disassembled by shear stress; when shearing forces were removed, the formulation regained its initial viscosity, in agreement with the results of Penzes et al.^28^. Furthermore, the results of the present study showed that terpinen-4-ol did not alter the activity of the formulations.

The results also showed that the addition of artificial saliva to the formulation decreased the flow rate and caused thixotropy, which is the phenomenon by which colloids change their viscosity. These thixotropic properties were directly related to the interactions between components in the formulation. Because of interaction forces between the more structured regions, this can be broken by increasing the shear rate, although this is easily recovered when the shearing force decreases^29^. When 100% artificial saliva was added to F and F100, pseudoplastic behavior and high viscosity were observed. In addition, F100 had a thixotropic system, indicating that the system was more structured in F100 than in F.

Mucoadhesion can be defined as the adhesion between a synthetic or natural material and surface of a tissue, particularly the mucosal epithelium. The function of the designed LCS was to extend the residence time at the site of action or absorption by increasing drug contact with the epithelial barrier of the skin or mucosa^30^. In this study, the LCS containing nystatin, terpinen-4-ol, and 100% artificial saliva exhibited the best bioadhesive properties. These results are consistent with those of Salmazi et al.^31^ and Aida et al.^27^, who showed that the addition of artificial saliva to the LCS increased bioadhesion. The results presented here show that the addition of mucoadhesive polymers such as chitosan can increase viscosity and mucoadhesive characteristics. Thus, the formulations developed may be clinically important because of their higher values of mucoadhesion, indicating a prolonged adhesion period and increased drug absorption. The increase in viscosity with increasing saliva concentrations was also described in the literature as a property that facilitates mucoadhesion in different mucous membranes^27,32^.

The mechanism of action of nystatin is to bind to steroids within the cell membranes of susceptible fungi, disrupting cell membrane permeability and causing cytoplasmic content to leak from the cell; however, nystatin displays several side effects in humans, including gastrointestinal problems and an unpleasant taste^33^. The topical use of nystatin is limited in terms of the period of localization, mainly because of the added flavoring agents that increase the volume of saliva in the area and thus reduce the time of residence at the injured site, reducing the effectiveness of treatment^34,35^. In contrast, terpinen-4-ol operates by disrupting the fungal cell membrane to interfere with the integrity and cell physiology of the microorganism. Our results agree with those of Ramage et al.^14^ and Tonon et al.^36^, who showed that terpinen-4-ol exhibits strong antimicrobial properties against planktonic cultures and fungal biofilms and may be suitable for the prophylaxis and treatment of candidiasis.

Antimicrobial agents as terpinen-4-ol reduce the proliferation of pathogens in general as well as oral pathogens^17,36^. In this context, the efficacy of the combination of terpinen-4-ol and nystatin has been demonstrated in vitro by Tonon et al.^36^, who demonstrated the importance of this compound against *Candida* species. However, several studies have shown that terpinen-4-ol inhibits bacterial growth and adhesion to dental biofilm^12,13,17^. A previous study reported that terpinen-4-ol 0.24% and 0.95% exhibited antimicrobial effects against *S. mutans* and *L. acidophilus* in both planktonic and biofilm forms^12^. Similarly, Bucci et al.^13^ observed the antiadhesion and antibiofilm properties of the mixture of surfactin and terpinen-4-ol against *S. mutans*, *P. gingivalis*, and *C. albicans*. Additionally, the effectiveness of terpinen-4-ol on periodontal pathogens has already been demonstrated and a concentration of 0.19% was found to be effective for reducing the number of microorganisms of single and multispecies biofilms^17^.

The current results showed that when nystatin and terpinen-4-ol were incorporated into the LCS, they had strong antimicrobial activity. When the drugs were incorporated into the LCS, antifungal and antibiofilm activities were increased compared to the drugs without LCS. Similarly, Salmazi et al.^31^ and Fonseca-Santos et al.^37^ developed, characterized, and evaluated an LCS incorporated with curcumin, and observed a greater effect against *C. albicans* than when the drug was applied without the system. This may be because the LCS has a strong binding affinity for lipids in the fungal cell wall and may increase interactions with the cell wall.

Exploiting the interactions between compounds is a novel approach for improving the antimicrobial efficacy of drugs and reducing the concentration required to control infections. The synergistic action of the components in this study was evaluated by the checkerboard test. This test analyzes the effect of combining different concentrations of each compound to verify the efficiency of their synergistic interactions^38^. Furthermore, it was demonstrated that the combination of terpinen-4-ol with nystatin was highly effective against *C. albicans* strains. In addition, the synergistic effect of these components was also observed when incorporated in the LCS. Nikolić et al.^39^ demonstrated the synergistic effects of essential oils from *Citrus limon*, *Piper nigrum* (green pepper), and *M. alternifolia*, and found that their combined use resulted in increased efficacy. Other studies have reported the successful combination of essential oils with conventional drugs, including nystatin^8,39^.

It is difficult to compare the results of this study with those of other investigations using the LCS, as several factors in the preparation of the LCS precursor, including concentration, fungal strains, and others, can influence the results in unpredictable manners. Considering the high capacity of fungi to develop resistance to antimicrobials, the current results demonstrated that the LCS precursor incorporated with terpinen-4-ol and nystatin for buccal administration is a promising alternative for preventing and treating oral candidiasis. In addition, considering the potent broad-spectrum microbicidal and antibiofilm activities of terpinen-4-ol, the development of the current LCS may be effective for eliminating also pathogenic bacteria. However, despite the promising results of this study, there were some limitations, and further studies are needed to confirm the effectiveness of LCS for oral applications. The use of compound interactions is a new approach for improving antimicrobial efficacy with a reduced effective concentration to control infections.

## Conclusion

We developed and characterized an LCS containing terpinen-4-ol and nystatin. PLM and rheological analyses showed that the LCS had antifungal activity and a synergistic/additive effect caused by incorporation of nystatin and terpinen-4-ol. Mucoadhesion analyses showed that addition of artificial saliva to the LCS improved its viscosity and mucoadhesive characteristics. Antifungal analysis showed that the LCS was more potent and effective against *C. albicans* strains compared to other treatments. Furthermore, our in vitro results demonstrated that the LCS is a promising alternative for the prevention and treatment of infections, and can be used to develop novel therapeutic strategies.

## Material and methods

### Solutions

Terpinen-4-ol (Sigma-Aldrich, St. Louis, MO, USA; CAS number: 20126-76-5) was prepared at concentrations ranging from 9.16 to 1.06 mg/mL Roswell Park Memorial Institute (RPMI 1640) medium and 0.4% dimethyl sulfoxide (DMSO) (Sigma-Aldrich, St. Louis, MO, USA) to increase the solubility of the oil. Nystatin solution (Sigma-Aldrich, St. Louis, MO, USA; CAS Number: 1400-61-9) was prepared in DMSO as a stock solution at a concentration of 0.064 mg/mL and subsequently diluted from 0.064 to 0.00012 mg/mL (RPMI-1640 medium, pH 7.0, 0.165 M MOPS, 2% glucose), according to the M27-A2 standard CLSI^40^.

### Development of the LCS

The system was composed of propoxylated and ethoxylated cetyl alcohol (PPG-5-CETETH-20) (Procetyl^®^ AWS), oleic acid (AO), and 0.5% chitosan dispersion (QS), and was prepared by the phase diagram method^26^. The aqueous phase of the system was prepared with a final polymer percentage of 0.5% QS. Subsequently, 54 different mixtures, at varying proportions from 0–100% (w/w), of each phase of the system were prepared, resulting in the construction of a 54-point ternary phase diagram. The formulations obtained were visually characterized in a liquid translucent system (STRL), a viscous translucent system (STRV), a liquid transparent system (STL), a viscous transparent system (STV), an opaque liquid system (SLO), an opaque viscous system, and by phase separation (SF). Terpinen-4-ol (Sigma Aldrich, St. Louis, MO, USA) was incorporated at 0.57–9.16 mg/mL into the oil phase of the system, as described previously^11^. Nystatin was incorporated at concentrations of 3.125 × 10^–5^–6.4 × 10^–2^ mg/mL, as described in document M27-A2 of the CLSI^40^. A volume of 1 L of artificial saliva was prepared by mixing 8.0 g of NaCl, 0.19 g of KH_2_PO_4_, and 2.38 g of Na_2_HPO_4_. After complete mixing, the pH was measured as 6.8^41^.

### Effect of artificial saliva on the formulations

The precursor of liquid crystalline system (FC), composed of 40% oil phase, 40% surfactant, and 20% water, was selected for the incorporation of terpinen-4-ol (FT), nystatin (FN), or both (FNT). To verify if the FC, FT, FN and FNT behaved like precursor of LCS, 30% (FC30, FT30, FN30, FNT30) and 100% (FC100, FT100, FN100, FNT100) of artificial saliva were added into FC, FT, FN and FNT. That is, in each 1.0 g of formulation (FC, FT, FN, FNT), 0.3 g of artificial saliva (FC30, FT30, FN30, FNT30) or 1.0 g of artificial saliva (FC100, FT100, FN100, FNT100) were added.

To investigate the effects of both artificial saliva and drugs in the formulation structures, polarized light microscopy (PLM), rheology, and mucoadhesion analyses were performed. The groups were as follows: LCS precursor formulation (FC), LCS precursor with terpinen-4-ol (FT), LCS precursor with nystatin (FN), and LCS precursor with nystatin and terpinen-4-ol (FNT), LCS precursor formulation (FC) with 30% or 100% artificial saliva (FC30 or FC100), LCS precursor with terpinen-4-ol (FT) with 30% or 100% artificial saliva (FT30 or FT100), LCS precursor with nystatin (FN) with 30% or 100% artificial saliva (FN30 or FN100), and LCS precursor with nystatin and terpinen-4-ol (FNT) with 30% or 100% artificial saliva (FNT30 or FNT100).

### Physicochemical characterization

#### Polarized light microscopy (PLM)

All formulations were applied (10 μL) on a glass slide and covered with a coverslip. Samples were observed by PLM (Olympus BX41, Tokyo, Japan) with a plug (QColor3 Olympus America, Inc.) at 20 × magnification and 25 °C. The samples were then analyzed for the presence of anisotropy or isotropy within the dispersion^29^.

#### Rheological analysis

Rheological measurements were performed at 37 ± 0.1 °C in triplicate using a controlled-stress AR2000 rheometer (TA Instruments, New Castle, DE, USA) with parallel plate geometry (diameter: 40 mm) and a sample gap of 200 μm. The samples from each formulation were carefully applied to the lower plate to minimize sample shearing and were allowed to equilibrate for 3 min prior to analysis^31^.

#### Determination of flow properties

The flow properties were determined using a shear rate of 0.01–100 s^−1^ for the ascending curve and 100–0 s^−1^ for the descending curve, for 120 s each at 10-s intervals. The consistency index and flow index was determined based on “Eq. (1)” for quantitative analysis of flow behavior

1 $$\tau \, = \,k\gamma \, \eta$$

where τ is shear stress, γ is shear rate, *k* is consistency index, and η is flow index^42^.

### Oscillatory analysis

Oscillatory analyses were initiated by performing a stress sweep to determine the viscoelastic region of each formulation. The stress sweep was carried out at a constant frequency of 1 Hz over a stress range of 0.1–10 Pa. Next, a constant shear stress of 1 Pa was selected to perform a frequency sweep over a range of 0.1–10 Hz, which was within the previously determined linear viscoelastic region for all formulations. Thus, the storage (G′) and loss (G″) moduli were recorded. The variations in G′ at low frequencies in a log–log plot of G″ versus ω followed the power law, as described in “Eq. (2)”:

2 $${\text{G}}\, = \,{\text{S}}.\omega^{{\text{n}}}$$

where S is the formulation strength, ω is the oscillation frequency, and n is the viscoelastic exponent^31^.

### In vitro evaluation of the mucoadhesive force

The in vitro mucoadhesive strength of the formulations, the strength required to remove the formulation from the swine esophageal mucosa was evaluated in vitro using a TA-XT plus texture analyzer (Stable Micro Systems, Surrey, England) in the adhesion test mode. First, swine esophageal mucosae were prepared according to Calixto et al.^20^ and the mucosa was immersed in artificial saliva for 30 s to simulate the buccal environment. The mucosa was then attached to the lower end of the cylindrical probe (10-mm diameter) and the formulations were then placed in a vial under the probe at 37 °C. Then, the probe came down at 1 mm/s until the mucosa touches the formulation. The time of contact was 60 s with no force applied. Thereafter, the probe come up at 0.5 mm/s until detachment of the mucosa from the formulation occurred. The force required for this detachment to occur was calculated from the force versus time curve^43^, performed in triplicate.

### Microbial strains and antimicrobial solutions

This study included clinical isolates of *C. albicans* (Genotype A and B) collected from patients with diabetes and chronic periodontitis. This study protocol was approved by the Ethical Committee of Research, Piracicaba Dental School, State University of Campinas (UNICAMP) (Protocol Nº 062/2008). All participants were informed and agreed to participate in the study. Individuals were submitted to a periodontal examination and sample collection. The samples were processed in the microbiology and immunology laboratory of the Piracicaba Dental School—UNICAMP. All experiments were performed in triplicate in three independent experiments^8^. *C. albicans* reference strain SC 5314 were also used in this study.

The *Candida* species were grown in RPMI 1640 medium supplemented with 2% glucose and 0.165 M MOPS (3-(*N*-morpholino) propanesulfonic acid at pH 7.0 and then incubated at 37 °C for 18 h. The suspensions were adjusted to a final concentration of 1.0 × 10^7^ CFU mL^−1^ using a spectrophotometer (Eppendorf AG, 22331, Hamburg, Germany). Nystatin solution (Sigma-Aldrich) was prepared in DMSO (Sigma-Aldrich) at concentration of 0.064 mg/mL and subsequently diluted. Terpinen-4-ol dilutions were prepared in RPMI 1640 medium and DMSO 0.4%. All subsequent experiments were performed in triplicate in three independent assays.

### Minimum inhibitory concentration (MIC) and minimum fungicidal concentration (MFC)

The broth microdilution technique (CLSI) was used to determinate the minimum inhibitory concentration (MIC). Serial dilutions of terpinen-4-ol and nystatin were added to 96-well plates. The fungal suspensions (1.0 × 10^3^ CFU mL^−1^) prepared in RPMI were added to each well and the plates were incubated at 37 °C and maintained with shaking at 75 rpm. *Candida* species without terpinen-4-ol and nystatin were used as a control group. A DMSO group was used to demonstrate that this agent did not interfere with the results. After 24 h the wells were evaluated visually and using an ELISA reader (OD = 492 nm). The MIC was defined as the lowest concentration of each drug able to completely inhibit growth according to spectrophotometry analysis.

Subsequently, the minimum fungicidal concentrations (MFC) were established. Briefly, 10 µL samples were withdrawn and plated on Sabouraud Dextrose Agar culture medium (Acumedia, Lansing, MI, USA) and the cultures were maintained at 37 °C for 48 h. The MFC was defined as the lowest concentration able to reduce *Candida* species by ≥ 99.9%. All experiments were performed in triplicate and in three independent experiments.

### Biofilm formation and vitality measurement by XTT assay

Biofilm formation was performed in 96-well microtiter plates, as described by Francisconi et al.^11^. The suspensions of *C. albicans* were adjusted to a final concentration of 1.0 × 10^3^ CFU mL^−1^ in RPMI medium and maintained at 37 °C for 48 h. The culture medium was removed and concentrations of terpinen-4-ol, nystatin, and LCS associated with test solutions were added to *C. albicans* biofilms. After 24 h, the resulting biofilms were washed with PBS and viability mensuration was performed using XTT reduction assay (2,3-bis-(2-methoxy-4-nitro-5-sulfophenyl)-2H-tetrazolium-5-carboxanilide) (Sigma-Aldrich, St. Louis, MO, USA), in which the cells metabolically reduce sodium to a soluble formazan product^44^. Next, the plates were incubated at 37 °C for 3 h and absorbance was measured at 492 nm using an ELISA reader (Multiskan, Ascent 354, Labsystems CE).

### Fractional inhibitory concentration index (FICI)

Planktonic microorganisms and biofilms were evaluated by the lowest concentrations of each drug able to completely inhibit growth. The CIMs values were converted to fractional inhibitory concentrations Index (FICI) using the “checkerboard” method. These are equivalent to the ratio of drugs MIC (A and B) combined with the MIC of each drug, as described below. Thus, the FICI was calculated as the sum of the fractional inhibitory concentrations of each drug used^45^.

$${\text{FICA }} = {\text{ MIC of drug A with B}}/{\text{MIC of drug A alone}}$$

$${\text{FICB }} = {\text{ MIC of drug B with A}}/{\text{MIC of drug B alone}}$$

$${\text{FICI }} = {\text{ FICA }} + {\text{ FICB}}$$

In this study, the FICI was interpreted to indicate synergism when 0.5, additive or indifferent when > 0.5 and ≤ 1, and antagonism when > 1^45,46^. All experiments were repeated in triplicate on three different days.

### Statistical analysis

The LCS precursor was characterized using Tukey's test. Comparisons of the means within individual groups were performed. Mucoadhesion force values were evaluated by ANOVA and Tukey’s test. For biofilm reduction analysis, analysis of variance and Kruskal–Wallis and Mann–Whitney tests were used. A value of *p* < 0.05 was considered as statistically significant.
